# Relationship Between Physical Activity, Cognition, and Emotional and Social Well-Being in Gifted Students: A Systematic Review

**DOI:** 10.3390/sports14050188

**Published:** 2026-05-06

**Authors:** Rubén Roldán-Roldán, Sara Suárez-Manzano, Alba Rusillo-Magdaleno, José Enrique Moral-García

**Affiliations:** 1 Research Group HUM-943, Faculty of Education Sciences, University of Jaén, 23071 Jaén, Spain; rrr00043@red.ujaen.es (R.R.-R.); jemoral@ujaen.es (J.E.M.-G.); 2 Institute for Transfer and Research (ITEI), Universidad Internacional de La Rioja (UNIR), 26006 Logroño, Spain; alba.rusillomagdaleno@unir.net

**Keywords:** physical activity, gifted students, executive functions, emotional well-being, cognitive development

## Abstract

Physical activity (PA) has been associated with improvements in cognitive function and psychological well-being in the school population; however, its specific impact on gifted students has been scarcely investigated. This systematic review analyzes the effects of PA, as well as the associations between PA and cognitive performance, emotional well-being, and social integration in students with high abilities (HA), also considering moderating variables such as sex and type of intervention. A total of four studies published between 2004 and 2025 were identified through searches in Web of Science, Scopus, and PubMed, including cross-sectional and quasi-experimental designs with more than 700 participants aged 8 to 13 years. The findings suggest that PA may be associated with improvements in memory, attention, and creativity, as well as lower levels of anxiety and stress. Benefits were also observed in body perception and social integration, although these effects appear to be influenced by factors such as gender and academic demands. Overall, the evidence suggests that PA may represent a potentially valuable educational resource for the comprehensive development of these students; however, further longitudinal and experimental studies are needed to establish more robust and context-sensitive intervention protocols.

## 1. Introduction

Students with high abilities (HA) are distinguished by exceptional potential in areas such as logical reasoning, creativity, memory, and complex problem solving [1]. Although there is no single, universally accepted definition, contemporary approaches agree that HA is a multidimensional phenomenon shaped by cognitive, emotional, and contextual factors [2]. Models such as Renzulli’s Three-Ring Conception of Giftedness [3] and Gagné’s differentiation between endowment and talent [4] have contributed to understanding how individual potential interacts with the educational environment.

In the Spanish context, current legislation recognizes diverse profiles of students with HA, including giftedness, single and multiple talents, and early development, all of which require individualized assessment and adaptive pedagogical approaches [5]. Previous research has shown that academic performance in this group is not solely determined by IQ, but also by environmental quality, available resources, and the adequacy of educational practices [6]. Insufficient personalized support may lead to demotivation, frustration, or social isolation [7].

Within this framework, physical activity (PA) has emerged as a relevant factor in holistic development. Previous studies suggest that regular PA practice may be associated with improvements in executive functions, working memory, sustained attention, and emotional self-regulation. At the neurobiological level, PA has been linked to enhanced neuronal connectivity, neurogenesis, and synaptic plasticity during key developmental stages [8]. Furthermore, its positive influence on psychological well-being—including improved self-concept, reduced anxiety, and a stronger sense of belonging—has been particularly relevant in populations with specific educational needs, such as students with HA [9,10].

However, empirical evidence regarding the relationship between PA and cognitive, emotional, and social outcomes in this population remains limited and fragmented. Some recent studies suggest that students with HA may engage in lower levels of PA, particularly girls, which could reduce their access to the associated benefits [11]. Factors such as academic pressure, high self-demand, and the lack of adapted programs may contribute to this pattern [12,13].

Despite international recommendations advocating at least 60 min of moderate-to-vigorous PA per day during childhood and adolescence [14], adherence to these guidelines remains insufficient. Moreover, it is still unclear to what extent PA may differentially affect students with HA [15]. Given this context, it is necessary to conduct a systematic analysis of the available evidence examining the relationship between PA and cognitive, emotional, and social variables in this population.

The objective of this systematic review is to identify and synthesize empirical evidence on the effects and associations of PA on cognitive performance, emotional well-being, and social integration in students with HA, while also considering moderating variables such as gender, age, and type of intervention. This review aims to provide evidence-informed guidance for the integration of PA into educational programs designed for this population from a holistic development perspective.

## 2. Materials and Methods

The study was conducted in accordance with the Preferred Reporting Items for Systematic Reviews and Meta-Analyses (PRISMA) guidelines [16] (Supplementary Materials). The review protocol was registered in PROSPERO under registration number CRD420251073268.

In line with Cochrane guidelines for systematic reviews of interventions, Table 1 presents the databases consulted, the search strategies applied, the search limits, and the number of records retrieved to ensure the reproducibility of the review process.

The same search strategy was applied across all databases (PubMed, Web of Science, and Scopus), with minor adaptations to database-specific syntax and indexing terms. The search strategy combined four conceptual domains (physical activity, cognition, giftedness, and school-age population) using nested Boolean operators (AND/OR).

### 2.1. Search Limits

A systematic search was conducted across three major bibliographic databases: PubMed, Web of Science, and Scopus. The search covered publications from January 2004 to December 2025. Additionally, the reference lists of the selected studies were manually screened to identify further relevant articles.

Four main groups of search terms were combined using Boolean operators:

1. Physical activity: “Physical activity”, “exercise”, “physical fitness”, “sports”, “aerobic exercise”, “motor skills”, “physical education”.
2. Cognition: “Cognition”, “cognitive skills”, “executive function”, “memory”, “attention”, “problem solving”, “creativity”, “thinking skills”.
3. Gifted children: “Gifted”, “gifted children”, “talented”, “intellectually gifted”, “high potential”.
4. School-age children: “Primary school”, “secondary school”, “adolescents”, “children”, “school-age youth”.

### 2.2. Selection Criteria

Articles included in this review were selected according to the following criteria: (1) the study was published as a full article in a peer-reviewed journal; (2) the study focused on students with high abilities (HA) or intellectually gifted students; (3) participants were aged between 6 and 18 years, corresponding to primary or secondary education; (4) the study was published in English, Spanish, or in another language with an available translation; (5) the study used validated instruments to assess the analyzed variables; and (6) the study explicitly measured physical activity, physical fitness, sports participation, exercise intervention, or movement-based programs as a primary independent variable.

### 2.3. Data Extraction and Reliability

The study selection and data extraction process was conducted independently by two reviewers (RRR and SSM), who screened the titles and abstracts of all identified studies. Any disagreements regarding study inclusion were resolved through discussion and consensus.

For each selected study, the following information was extracted: authors and year of publication, study objective, sample size, participants’ age, country of origin, study design, measurement of physical activity, assessment of giftedness, confounding factors considered, and the main outcomes and conclusions.

### 2.4. Assessment of Quality and Level of Evidence

The quality assessment was conducted based on standardized assessment criteria and according to the selection criteria established in this review. The checklist included six items: (A) whether the study was published as a full report in a peer-reviewed journal; (B) whether the study focused on students with HA; (C) whether PA, cognitive, and behavioral outcomes were clearly described; (D) whether the population included participants aged between 6 and 18 years, corresponding to primary and secondary education; (E) whether the study used a cross-sectional, longitudinal, or experimental design; and (F) whether the analyses controlled for potential confounding factors.

Each item was rated as 2 (fully reported), 1 (partially reported), or 0 (not reported or unclear). A total quality score was calculated by summing the scores of all items, resulting in a possible range from 0 to 12. The characteristics and quality assessment of the included studies are presented in Table 2. Based on the total score, three levels of methodological quality were established: high quality (HQ), defined as scores between 9 and 12; medium quality (MQ), defined as scores between 5 and 8; and low quality (LQ), defined as scores below 5.

After the quality assessment, most included studies were classified as high quality (HQ). However, this classification should be interpreted with caution given the heterogeneity in study designs, sample sizes, and measurement procedures. In particular, some studies present a higher risk of bias due to the reliance on self-reported measures, small sample sizes, and the absence of control groups. Therefore, the quality assessment was used as a general methodological reference rather than as a strict comparative indicator across studies.

## 3. Results

### 3.1. General Findings

**Figure 1:**
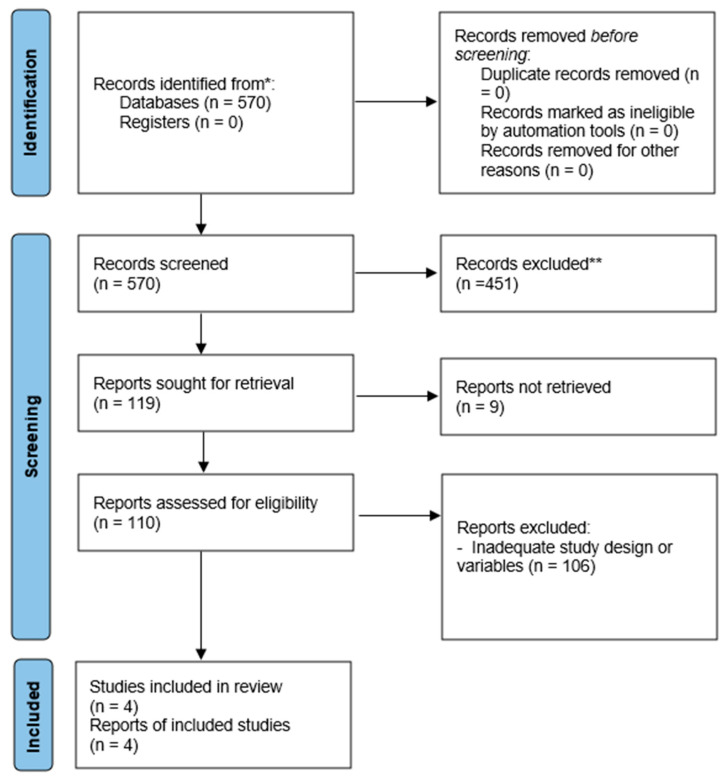
PRISMA-based flowchart. * Records identified from databases. ** Records excluded during the screening phase based on the predefined inclusion and exclusion criteria.

The flow of search results through the systematic review process is presented in Figure 1. Initially, a total of 570 records were identified across the databases, including PubMed (*n* = 91), Web of Science *(n* = 269), and Scopus (*n* = 208). Additionally, two records were identified through other sources.

During the initial screening, 451 records were excluded because they did not meet the established inclusion criteria (population, age, or language). Subsequently, nine duplicate records were removed, leaving 110 articles for full-text assessment. During the eligibility phase, 106 studies were excluded because they did not meet the defined design criteria or variables.

Finally, four studies met all eligibility criteria and were included in the review. The main characteristics of the included studies are summarized in Table 3.

### 3.2. Relationship Between Physical Activity and Cognition, Emotional State and Motor Skills (Acute Effects and Cross-Sectional Associations)

Several cross-sectional studies and brief interventions have explored the relationship between PA and cognitive, emotional, and behavioral variables in students with HA.

In an action research study, Ford [18] investigated the effects of a daily 20-min moderate aerobic PA intervention delivered over four weeks on memory and emotional state in 16 gifted schoolchildren (4 boys and 12 girls, aged 9 to 11 years). The results indicated statistically significant improvements in declarative memory (*p* = 0.05) and a subjective reduction in stress in 87.5% of participants (*p* = 0.01), suggesting the short-term positive effect of PA on cognitive and emotional functions. However, the small sample size and the absence of a control group limit the generalizability of these findings.

Akgül [19] conducted a cross-sectional observational study with 199 gifted schoolchildren (aged 8 to 13 years), analyzing self-reported PA levels in relation to mental health indicators. The findings revealed an inverse association between PA and anxiety levels, particularly in boys (β = −0.16, *p* = 0.001). In addition, greater time spent playing digital games was associated with higher anxiety levels, while resilience showed a negative association with anxiety. These results support the hypothesis that regular PA may have a protective role against emotional symptoms in this population. Nevertheless, the reliance on self-reported measures may introduce measurement bias.

In the study conducted by Otero Rodríguez et al. [11], using a cross-sectional design, PA was assessed through the IPAQ-SF questionnaire and physical fitness tests in 148 schoolchildren (74 with HA and 74 in the control group). Although no significant objective differences in physical fitness were observed between groups, girls with HA reported lower PA levels but greater satisfaction with their body image. Although no direct cognitive measures were included, the authors suggested that positive body perception may contribute to emotional and social well-being, which are relevant dimensions in the education of students with HA.

Taken together, these studies suggest that even moderate or self-reported levels of PA may be associated with benefits in cognitive, emotional, and perceptual domains. However, due to the cross-sectional designs, reliance on self-reported measures, and short-term nature of interventions, causal relationships cannot be established. This highlights the need for more rigorous longitudinal and experimental research to clarify these associations.

### 3.3. Sustained Effects of Physical Activity on Cognition and Well-Being in Gifted Students

Studies examining the influence of PA through prolonged interventions or quasi-experimental designs suggest potential beneficial long-term effects, particularly on complex cognitive processes such as creativity and aspects of psychological well-being.

Memmert (2006) [17] evaluated the impact of a weekly physical enrichment program focused on creative play and motor problem-solving over a six-month period in 33 schoolchildren (18 with HA and 15 without HA), with a mean age of 8.2 years. The experimental group showed significant improvements in tactical creativity tasks, particularly in originality and flexibility. The group × time interaction was significant (*p* = 0.05), indicating that the observed change was attributable to the intervention rather than to maturational development. However, the relatively small sample size limits the external validity of these findings.

Overall, studies using long-term or quasi-experimental designs suggest that sustained PA may be associated with improvements in advanced cognitive functions and aspects of psychological well-being in gifted students. Nevertheless, the available evidence remains limited in number and characterized by methodological heterogeneity, highlighting the need for broader longitudinal research with appropriate control groups and standardized assessment protocols.

## 4. Discussion

The findings of this systematic review provide a structured overview of the potential role of PA in the cognitive, emotional, and social development of students with HA. Although the scientific literature specifically focused on this population remains limited, the included studies suggest that PA may be associated with benefits in executive functions, psychological well-being, and body perception, which are relevant dimensions of holistic education.

An important distinction concerns the methodological design of the analyzed studies. While some investigations explored associations using cross-sectional approaches [11,19], others examined the effects of short- or long-term PA interventions [17,18]. This distinction is essential when interpreting the findings and determining the extent to which causal inferences can be made.

Regarding short-term effects, Ford [18] reported that a daily intervention consisting of 20 min of aerobic PA over four weeks improved declarative memory and reduced perceived stress in a small sample of schoolchildren with HA. Although these findings are promising, they should be interpreted cautiously due to the limited sample size. Similarly, Akgül [19], through a cross-sectional study, identified an inverse relationship between self-reported PA levels and anxiety, particularly among boys. However, because of the observational design, these findings only support associative rather than causal interpretations.

In the study conducted by Otero Rodríguez et al. [11], also using a cross-sectional design, lower levels of PA were observed among girls with HA, although they reported greater satisfaction with their body image compared to the control group. Although no direct cognitive indicators were assessed, these findings suggest that positive body perception may contribute to self-concept and emotional well-being. The lower participation of girls in PA may reflect sociocultural influences, academic self-demand, or motivational climates that deserve greater attention in future interventions.

With regard to longer-term effects, Memmert (2006) [17] implemented a six-month sports enrichment program focused on creative play and motor problem-solving, observing improvements in originality and flexibility, which are components of divergent thinking. These findings suggest that structured PA interventions may contribute to higher-order cognitive processes in students with HA.

From a social perspective, the available evidence suggests that PA may be associated with improvements in social integration in students with HA, as well as with cooperation, emotional regulation, and a sense of belonging. These aspects are particularly relevant because this population may experience interpersonal adjustment difficulties in conventional school settings [10,20]. Therefore, adapting PA experiences to their cognitive and emotional profiles may contribute to inclusion as well as to broader developmental needs.

Overall, the available evidence suggests that PA, when appropriately integrated into educational settings, may represent a potentially valuable pedagogical resource for addressing the needs of students with HA. Nevertheless, these conclusions should be considered preliminary due to the methodological limitations present in many of the reviewed studies. Further longitudinal and experimental research with greater methodological rigor is needed to confirm these associations and clarify the mechanisms involved.

### Limitations and Recommendations for Future Research

Despite these promising findings, this review presents several limitations. The scarcity of studies specifically focused on students with HA, together with the predominance of small and relatively homogeneous samples, limits the generalizability of the findings. Additionally, some included studies present a higher risk of bias due to the absence of control groups and small sample sizes.

In addition, a substantial proportion of the evidence is based on self-reported PA measures. The limited use of objective assessment tools, such as accelerometers, may reduce measurement precision and increase perceptual bias. Future studies should therefore prioritize standardized instruments and objective measures of both PA and cognitive performance [8].

Another limitation concerns the lack of diversity in analyzed samples. Most studies have focused on urban populations with medium to high socioeconomic status, which restricts applicability to more diverse educational contexts. Future research should incorporate variables such as gender, socioeconomic status, and cultural background to better understand individual differences in responses to PA.

Longitudinal studies with well-defined control groups are also essential to evaluate sustained effects of PA on cognitive and emotional outcomes. Current evidence suggests that longer interventions may have stronger effects on complex cognitive processes; however, such designs remain scarce in research involving students with HA.

From a practical educational perspective, future interventions could benefit from inclusive pedagogical frameworks that intentionally integrate movement with cognitive stimulation. Models centered on body-cognition integration may help schools design PA experiences that simultaneously support learning, emotional regulation, and social participation.

Finally, the integration of educational technologies may open new possibilities for personalizing PA programs aimed at students with HA. Digital resources designed with a pedagogical approach could improve motivation, monitoring, and adaptation of physical learning processes [21], thereby contributing to more inclusive and effective educational practices.

In conclusion, promoting research based on rigorous methodological designs and pedagogical approaches sensitive to the characteristics of students with HA is essential. Only through this type of research will it be possible to better understand how PA may support the cognitive, emotional, and social development of this population.

## 5. Conclusions

This systematic review suggests that PA may represent a potentially valuable pedagogical resource for supporting the cognitive, emotional, and social development of students with HA; however, this interpretation should be considered in light of the limited number of studies and their methodological heterogeneity. Among the most consistent findings identified are potential benefits in executive functions related to learning, such as memory, sustained attention, and creativity, as well as associations with lower levels of anxiety and stress, which are relevant aspects of psychological well-being in this population.

In addition, PA may be associated with aspects of social integration, including self-esteem, cooperation, and positive peer interactions. Although such patterns have been observed in both acute and longer-term interventions, sustained programs may offer opportunities to reinforce healthy lifestyle habits and support adaptive developmental processes.

However, the methodological limitations of the reviewed studies, including cross-sectional designs, small sample sizes, reliance on self-reported measures, and limited contextual diversity, restrict the generalizability of these findings. Therefore, the current evidence should be interpreted as preliminary.

In this context, designing PA programs adapted to the characteristics of students with HA remains an important educational challenge. Such interventions should consider individual variables such as age, cognitive and emotional profiles, as well as family and school environments. The integration of educational technologies together with active and inclusive teaching methodologies may further enhance the accessibility and personalization of these interventions.

Overall, the inclusion of PA within educational programs for students with HA may represent a promising strategy for supporting learning, mental health, and holistic development. Nevertheless, these findings should be interpreted with caution given the limited number of studies and their methodological variability. Stronger longitudinal and experimental evidence is still required before establishing definitive educational recommendations.

## Figures and Tables

**Table 1:** Database search strategy.

Database	Search Strategy	Limits	Filter
PubMed	(“physical activity”[Title/Abstract] OR “exercise”[Title/Abstract] OR “physical fitness”[Title/Abstract] OR “sports”[Title/Abstract] OR “aerobic exercise”[Title/Abstract] OR “motor skills”[Title/Abstract] OR “physical education”[Title/Abstract]) AND (“cognition”[Title/Abstract] OR “cognitive skills”[Title/Abstract] OR “executive function”[Title/Abstract] OR “memory”[Title/Abstract] OR “attention”[Title/Abstract] OR “problem solving”[Title/Abstract] OR “creativity”[Title/Abstract] OR “thinking skills”[Title/Abstract]) AND (“gifted”[Title/Abstract] OR “gifted children”[Title/Abstract] OR “intellectually gifted”[Title/Abstract] OR “high potential”[Title/Abstract]) AND (“children”[Title/Abstract] OR “adolescents”[Title/Abstract] OR “school-age youth”[Title/Abstract] OR “primary school”[Title/Abstract] OR “secondary school”[Title/Abstract])	Publication date from 1 January 2004 to 1 December 2025 -Species: Humans Age: 6–12 and 13–18 -Language: All languages	14 articles filtered
Web of Science			51 items filtered
Scopus			51 items filtered

**Table 2:** List of included studies with quality scores.

Authors and Variables	A	B	C	D	E	F	Total Score	Quality Score
Memmert [17]/Giftedness, creative thinking, sports enrichment and non-gifted children	2	2	2	2	2	2	12	HQ
Ford [18] /Aerobic activity, stress, memory, gifted students.	2	2	2	2	2	0	10	HQ
Otero Rodríguez et al. [11] Physical activity, physical fitness, body image in gifted children	2	2	2	2	2	1	11	HQ
Akgül G. [19] Resilience, physical activity, digital games and anxiety in gifted children during the pandemic	2	2	2	2	2	1	11	HQ

Note: High quality (HQ) = 9–12. Although all included studies reached scores within the high-quality range according to the applied checklist, this classification should be interpreted with caution. The tool used may not fully differentiate between study designs with different levels of evidential strength. In particular, some studies present a higher risk of bias due to small sample sizes, reliance on self-reported measures, and the absence of control groups.

**Table 3:** Characteristics of the studies analyzed (*N* = 4).

Authors/ Variables	Study Design/ Intervention (Duration)/ Confounding Factors	Sample/ Age (Years)/ Country	Groups/ Physical Activity/Intensity Measures	Cognition Measurement	Results
Memmert [17]	Field Study/Intervention: 6 months/Confounders: Age, Training Time, School Sports Participation	33 children (18 gifted, 15 not)/8.2 years/Germany	2 groups: GA (gifted, enrichment program), GC (non-gifted)/No objective measure of PA	Creative Thinking: Originality and flexibility in the sports game testing situation	GA showed creative improvement (*p* = 0.06); CG without improvement (*p* = 0.10); significant interaction (*p* = 0.05)
Ford [18]	Action research/Intervention: 20 min daily for 4 weeks/Confounders: age, giftedness, emotional state	16 students (4 boys, 12 girls)/9–11 years old/USA	1 gifted group/aerobic PA (20 min) before memory tasks	Memory Tests: Evaluation of Declarative Memory	Improvement in memory (*p* = 0.05); 87.5% reported less stress (*p* = 0.01); qualitative improvements in motivation and well-being
Otero Rodríguez et al. [11]	Observational/Confounders: age, gender, socioeconomic status, type of school	148 children (74 gifted, 74 not)/11.6 years/Spain	Gifted and non-gifted groups/Self-reported PA (IPAQ-SF)	Physical fitness: tests of flexibility, strength and aptitude; Body image: Stunkard pictogram adapted for young people	Significant differences in PA levels between gifted and non-gifted boys, with gifted girls being less active; Gifted children are more satisfied with body image
Akgül [19]	Cross-sectional observational/Confounders: gender, resilience, activity type	199 gifted/8–13 years old/Turkey	Self-reported PA, digital gaming	Anxiety: Spielberger State Anxiety Inventory (Likert scale), Resilience: Brief Resilience Scale	PA associated with lower anxiety in children (*p* = 0.001); Negative association between resilience and anxiety (*p* = 0.001)

## Data Availability

The data presented in this study are available within the article. No new data were created or analyzed in this study.

## Supplementary Materials

The following supporting information can be downloaded at: https://www.mdpi.com/article/10.3390/sports14050188/s1, PRISMA 2020 checklist. Reference [16] is cited in the supplementary materials.
